# Junior doctor strikes in South Korea: more doctors are needed?

**DOI:** 10.1016/j.lanwpc.2024.101056

**Published:** 2024-03-28

**Authors:** The Lancet Regional Health – Western Pacific

On Feb 1, 2024, the South Korean Government released a policy package to improve the performance of essential health-care services in the country. As part of the policy package, the Government plans to increase annual medical school admissions, starting in 2025, to address the shortage of doctors in rural areas and essential medical fields. This decision triggered a massive protest from the medical community. The Korean Medical Association, which represents about two-thirds of doctors in South Korea, argued that having more medical students would not be a solution to address the doctor shortage in essential medical care and would have a negative impact on medical education and patient care. Doctors, especially residents and interns, strongly opposed the decision and started to submit resignations on Feb 19, 2024; by March 8, 2024, more than 11,000 junior doctors were on strike. The health-care system has been disrupted due to this walkout as surgeries and emergency care are cancelled or postponed.

The South Korean Government has been attempting to lift the cap on medical student admissions since 2006 to address the shortage of doctors, but has been pushed back by health-care workers, including a monthly strike in 2020 during the COVID-19 pandemic. Although the number of doctors is increasing in South Korea, according to the *Health at a Glance 2023* report released by the Organization for Economic Cooperation and Development (OECD), South Korea has the lowest ratio of doctors per 1000 people compared with other high-income OECD countries. Physicians in South Korea generally earn more than average, but there have been long-standing concerns about working conditions and compensation for physicians providing essential medical care. Besides, essential medical departments deal with more life-and-death related health care, and doctors in these specialities have a higher risk of litigation.

There are mismatches between health needs and the medical manpower available in the country. The doctor shortage is not universal but is mainly in essential medical departments like paediatrics and emergency care. Medical resources and manpower are concentrated in the capital and urban areas, leading to regional disparities in health workforce distribution. The number of doctors per 1000 people in Seoul was 4.7, comparable with other high-income countries, while people living in non-capital areas have less than half this number of doctors. According to a statement from The Korean Pediatric Society, 12.5% of teaching hospitals in Seoul had no paediatric residents in 2022, and in other areas, the number can be as high as 20%. Physician shortage disrupts the medical system and harms the health of people in South Korea. From 2018 to 2022, there were about 37,000 patients who couldn’t get access to emergency care immediately; around 30% of these cases were caused by the lack of relevant physicians.

The reasons behind these disparities are deeply rooted in the medical system. South Korea’s health-care system is dominated by private providers and paid through a fee-for-service system by the national health insurance programme. Physician’s incomes are largely dependent on the volume of service they provide at a relatively low price decided by the national health insurance programme. The fee-for-service payment method and low medical fees create an incentive system to increase the volume of health services, which puts considerable pressure on the health-care system and ends up with longer wait times, shorter consultations, and prolonged working hours. Compared with eight other countries and areas including Australia, Belgium, Canada, France, Germany, Japan, and the USA, South Korea has the highest number of consultations per doctor, and is among countries and areas with the shortest consultation time. These factors affect physicians’ job satisfaction and harm doctor–patient relationships. Without further reform of the current health policy, the newly recruited medical students are mostly likely to flow into specialities with better payment and working conditions.

South Korea has one of the fastest ageing populations; non-communicable diseases (NCDs) will continue to be the leading causes of death in the country by 2040 based on a forecast study published in *The* *Lancet Public Health*. Like many other countries, there will be an increase in medical demands due to population ageing and rising burdens of NCDs. Under the current health-care delivery model, where large hospitals compete for more patients and patients have the freedom to choose their health-care providers, disparities and shortages of medical staff will be more complicated and further exacerbated due to changes in demographics and the disease spectrum.

The Government has been working on health reform to normalise medical fees for essential medical care, reduce physician’s liability in medical accidents, and enhance medical care in under-served areas. Systematic challenges exist in South Korea's health system that won’t be easily solved by having more doctors. The dispute between the Government and the medical community reflects the long-standing distrust between the two parties. Rather than taking legal action and revoking medical licenses, the Government and the medical community should search for other better options to avert a preventable public health crisis and to rebuild a trustful relationship between them to provide better health care for all Koreans.

